# Anisotropic Melting Path of Charge‐Ordering Insulator in LSMO/STO Superlattice

**DOI:** 10.1002/advs.202203933

**Published:** 2022-12-03

**Authors:** Bangmin Zhang, Ping Yang, Jun Ding, Jingsheng Chen, Gan Moog Chow

**Affiliations:** ^1^ Guangdong Provincial Key Laboratory of Magnetoelectric Physics and Devices Centre for Physical Mechanics and Biophysics School of Physics Sun Yat‐sen University Guangzhou 510275 China; ^2^ Singapore Synchrotron Light Source (SSLS) National University of Singapore 5 Research Link Singapore 117603 Singapore; ^3^ Department of Materials Science & Engineering National University of Singapore 9 Engineering Drive 1 Singapore 117576 Singapore

**Keywords:** anisotropic melting, charge‐ordering, manganite, phase diagram, spin‐orbital coupling

## Abstract

Multiple phases coexist in manganite with simultaneously active couplings, and the transition among them depends on the relative intensities of different interactions. However, the melting path with variable intensities is unclear. The concentration and the ordering of oxygen vacancy in previous work are found to induce ferromagnetic charge‐ordering insulator phase in [(La_0.7_Sr_0.3_MnO_3_)_10_/(SrTiO_3_)_5_]_n_ superlattice, which translates into metallic phase with magnetic field ***H*** and temperature ***T***. In the current work, the ***H***‐***T*** phase diagram for current ***I***//[100] and ***I***//[110] shows a large difference with ***H*** normal to the film plane, which is ascribed to the response of a variable range of hopping process to ***H*** with the in‐plane anisotropic hopping probability of charge carrier. With ***H*** rotating from the out‐of‐plane to the in‐plane direction, the preferred occupancy of the $3d_{z^{2}-r^{2}}$ orbital causes a decrease of spin‐orbital coupling and lowers the activation energy, inducing a gentler melting process of a charge‐ordering insulator. This work shows that the melting path of a charge‐ordering insulator phase can be largely modulated in manganite with anisotropy.

## Graphical abstract

*H*‐***T*** phase diagram of charge‐ordering phase in [(La_0.7_Sr_0.3_MnO_3_)_10_/(SrTiO_3_)_5_]_n_ superlattice shows large in‐plane anisotropy with ***H*** normal to film plane, and with ***H*** rotating from out‐of‐plane to in‐plane direction, the melting of this phase becomes gentler, which are due to the response of a variable range of hopping process to ***H*** with the in‐plane anisotropic hopping probability of charge carrier.

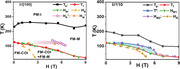

## 1 Introduction

Transition metal oxides^[^ ^1^ ^]^ possesses fascinating properties such as colossal magnetoresistance, superconductivity, and Berry curvature,^[^ ^2^ ^]^ due to the simultaneously active factors^[^ ^3^, ^4^ ^]^ including charge, lattice, orbital and spin. In manganite, multiple couplings,^[^ ^5^, ^6^ ^]^ such as double‐exchange, super‐exchange, and electron‐phonon coupling, have been proposed to describe the appearance of ferromagnetic‐metallic (FM‐M), ferromagnetic‐insulator^[^ ^7^ ^]^ (FM‐I) antiferromagnetic insulating (AFM‐I) and charge‐ordering insulating (COI) phases. Specially, the ferromagnetic charge‐ordering insulator (FM‐COI), could coexist with other phases in the frame of double‐exchange with impurity randomness^[^ ^8^ ^]^ in doped manganite, which is sensitive to magnetic field ***H*** and temperature ***T***. With the kinetic energy of the charge carriers dominates over the COI instability,^[^ ^9^ ^]^ the COI was proposed to melt and become a FM‐M or paramagnetic insulator (PM‐I), and the anisotropic spin‐orbital coupling (SOC) and orbital occupancy^[^ ^3^, ^10^ ^]^ could play an important role in this process. To date, the melting process of COI phase under external stimulus remains unclear and warrants further study.

The ordering of oxygen vacancy characterized by combined techniques,^[^ ^11^ ^]^ was presented to induce the FM‐COI phase in wideband manganite La_0.7_Sr_0.3_MnO_3_, which was susceptible to ***H*** and ***T***. The Mott's variable‐range hopping (VRH)^[^ ^12^ ^]^ model was proposed to explain the transport properties in the COI phase. At low ***H***, the resistivity decreases due to the quantum interference^[^ ^13^ ^]^ between paths connecting impurity sites during the hopping process; with further increasing ***H***, the field‐induced spatial shrinkage of electronic orbital would increase the resistivity. At the same time, strong ***H*** tends to enhance the hopping probability of the charge carrier along the direction of ***H***, which depends on the electronic orbital distribution and SOC intensity, and even could induce the collapse of the high resistivity COI phase.^[^ ^14^, ^15^ ^]^ For an epitaxial film with anisotropy of crystal structure, the intensities of different couplings might vary with the spatial direction and then affect the transition process of COI phase under the external stimulus.^[^ ^4^ ^]^

In this work, the La_0.7_Sr_0.3_MnO_3_/SrTiO_3_ (LSMO/STO) superlattice are fabricated with [LSMO_10_/STO_5_]_n_ configuration (SL_n_) on (001) LaAlO_3_ (LAO) substrate, 10‐unit cell (UC) LSMO and 5 UC STO in each period, and ***n*** is the number of periods. For both SL_2_ and SL_5_, the ***H***‐***T*** phase diagram shows large anisotropy for current with ***H*** normal to the film plane, ***I***//[100] and ***I***//[110]. The high resistivity the COI phase, which is sensitive to the strain status of the superlattice, was melted into a low resistivity phase with multiple steps (sharp change of resistivity). There is one more step for ***I***//[110] compared to that for ***I***//[100], which is ascribed to the response of VRH process to high ***H*** with in‐plane anisotropic hopping probability of charge carrier. In addition, ***H*** could lower the activation energy and increase the hopping distance, which makes the melting process gentler. With ***H*** rotating from the out‐of‐plane to the in‐plane direction, the preferred occupancy of $3d_{z^{2}-r^{2}}$ causes a decrease of SOC and lowers the activation energy, thus inducing a gentler melting process as observed in the magnetoresistance measurement. This work indicates that the melting path of COI phase could be largely modulated by changing the strength of different couplings.

## 2 Experimental Section

[LSMO_10_/STO_5_]_n_ superlattices (SL) were grown on (001) LAO substrate at 940 °C by pulsed laser deposition, the oxygen pressure was 1 mTorr for STO growth and 100 mTorr for LSMO growth. After deposition, the sample was cooled down at 15 °C /min in a 100 mTorr oxygen atmosphere. The appropriate oxygen pressure during STO growth and the existence of STO with larger out‐of‐plane lattice constant was proposed to promote the preferred location of oxygen vacancy on the (001) La/SrO plane in LSMO layer,^[^ ^11^ ^]^ which was crucial for the formation of the FM‐COI phase. For comparison, 50 UC and 20 UC LSMO single layers were also deposited on LAO substrate. There was no sign of the FM‐COI phase in Figure S1, Supporting Information, which supports that the STO layer is important for the formation of FM‐COI phase in SLs. The crystallographic property of the films at room temperature was studied using a four‐circle diffractometer (Huber 4‐circle system 90 000–0216/0) at the Singapore Synchrotron Light Source (SSLS), with X‐ray wavelength equivalent to Cu *K_*α*_*_1_ radiation. The magnetic properties were measured by a superconducting quantum interference device, and the transport properties were measured by the Physical Property Measurement System (PPMS) using linear four‐point probe.

## 3 Results and Discussion

**Figure 1:**
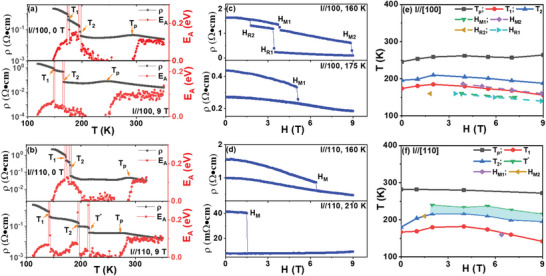
The resistivity‐temperature curve and calculated activation energy‐temperature curve (only the positive part) for SL_2_ on (001) LAO substrate, under different magnetic fields with a) I//[100] and b) I//[110]; the magnetoresistance curves at different temperatures with c) I//[100] and d) I//[110]; summarized magnetic field‐temperature phase diagram with e) I//[100] and f) I//[110]. The magnetic field is along out‐of‐plane directions.

**Figure** **1** shows the magnetoresistance (MR) and resistivity‐temperature (*ρ*‐T) curves for SL_2_. The current ***I*** was along in‐plane [100] and [110] direction, respectively, and the ***H*** was along the out‐of‐plane [001] direction. The *ρ*‐T curves were measured during the warming process with different ***H*** after zero‐field cooling from 300 to 10 K. The thermomagnetic measurement and magnetic hysteresis loop at low temperature in Figure S2, Supporting Information show that SLs are ferromagnetic below Curie temperature ***T***_C_. With increasing ***T***, there is two sharp down‐turn of resistivity (***T***_1_ and ***T***_2_) with ***I***//[100], indicating the melting of COI phase^[^ ^15^, ^16^, ^17^ ^]^ in LSMO layer into metallic phase; with ***H*** up to 9 T, the melting of COI phase was boosted with decreasing melting temperature. Similar behavior is observed for *ρ*‐T curves with ***I***//[110] in Figure 1b, but there is one additional sharp down‐turn (***T*** ^*^) with ***H*** = 9 T. In order to get more information about these multiple jumps in resistivity, MR curves at different ***T*** were measured with ***H*** along out‐of‐plane direction after zero‐field cooling from room temperature to 10 K and then back to targeted ***T***. Figure 1c,d shows typical MR curves, which also shows melting of COI phase with sharp down‐turn of resistivity. Both the temperature and magnetic field could melt the COI phase, and the summarized ***H‐T*** phase diagram is shown in Figure 1e,f. With increasing ***H***, one additional phase transition (***T*** ^*^) was induced with ***I***//[110], the origin of which would be the focus of the following study.

In the manganite COI phase, the Mott's VRH model was proposed to describe the transport properties in low temperature,^[^ ^12^ ^]^ $\rho =\rho_{0}\exp{\left(\frac{T_{0}}{T}\right)}^{1/4}$, and the activation energy ***E***_A_ required to induce the hopping^[^ ^18^ ^]^ could be obtained by $E_{A}=-\frac{K_{B}T^{2}}{\rho }\left(\frac{d\rho }{dT}\right)$, *K_B_* is the Boltzmann constant. Other model about the transport process are discussed in Figure S3, Supporting Information, indicating that the VRH could better describe the phenomenon. The calculated ***E***_A_ (red curve) shows obvious temperature dependence in Figure 1a‐,b at low ***T*** (only positive value was shown). The nonzero slopes of ***E***_A_‐T curve were the signature of variable range hopping,^[^ ^19^ ^]^ and the anomaly corresponds to the charge‐ordering transition. The constant ***E***_A_ above ***T***_p_ indicates that transport is dominated by the nearest neighboring hopping process at high ***T***, of which the activation energy does not present temperature dependence.^[^ ^20^ ^]^ From the ***E_A_‐T*** curve for ***H*** = 0 with ***I***//[100], there are two close anomalies (sharp jump out of the range of figure), corresponding to two sharp down‐turns in *ρ*‐T curve, and the ***E_A_*** value^[^ ^21^ ^]^ around these two anomalies are also close, indicating that the transport process should be similar. In addition, the magnetic measurement does not show any features around ***T***_1_ and ***T***_2_. Based on these experimental results, the ***T***_1_ and ***T***_2_ should be due to the spatial inhomogeneity in the film: the creation of percolative paths (such as metallic bridge between neighboring cluster^[^ ^22^ ^]^) at different places requires different temperatures and thus induce multiple jumps in the *ρ*‐T curve.^[^ ^23^ ^]^ For ***I***//[110], the shape of ***E_A_‐T*** curve with 9 T field changes largely compared to that without ***H***, showing three anomalies (***T***_1,_ ***T***_2,_ and ***T*** ^*^), which indicates that the magnetic field affects the transport process/mechanism. Considering the same direction of ***H***, the difference in transport behavior between ***I***//[110] and ***I***//[100], should come from the anisotropic‐conducting environment in the film plane, rather than additional phase transition, which would be discussed below.

**Figure 2:**
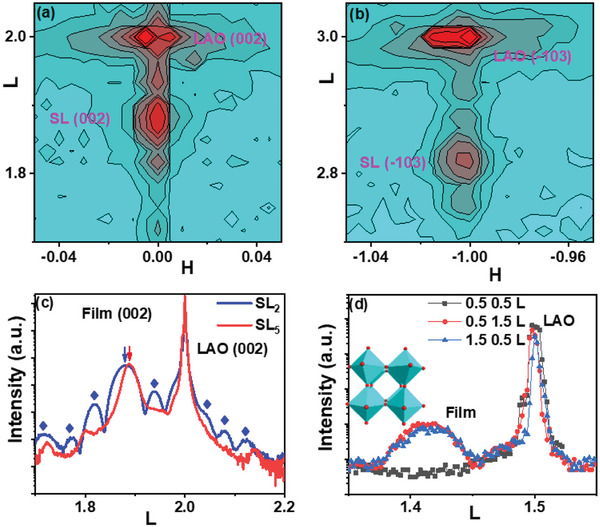
a) (002) RSM and b) (‐103) RSM for SL_2_; c) the ***L*** scan around (002) peak for SL_2_ and SL_5_; the arrow and the diamond (♦) indicate the main peak and satellites of the superlattice, respectively. d) the half‐integer diffraction for SL_2_, and the inset is the illustration of MnO_6_ ***a*** ^0^ ***a*** ^0^ ***c*** ^−^ pattern.

With a strong correlation between materials properties and crystal structure,^[^ ^24^ ^]^ the crystal structure of SLs was analyzed in **Figure** **2**. The *L* scan shows the (002) main peak and satellite peaks, labeled as arrow and diamond, respectively, indicating the formation of superlattice structure. The SL_2_ was fully strained by the (001) LAO substrate according to the (002) and (−103) RSM, and the out‐of‐plane lattice (***c*** = 4.029 Å) was elongated with compressed in‐plane lattice constant (***a*** = 3.790 Å) compared to that of bulk LSMO (3.880 Å); In addition, the oxygen octahedral rotation shows ***a*** ^0^ ***a*** ^0^ ***c*** ^−^ pattern^[^ ^25^ ^]^ according to the half‐inter diffraction in Figure 2d. The measured crystal structure shows the high quality of superlattice, which rules out the possibility that the multiple jumps in *ρ*‐T curves origins from the non‐uniformity. In order to get more correlation between the crystal structure and the hierarchy in ***H‐T*** phase diagram, the strain conditions of superlattice SL_5_ with ***n*** = 5 were also shown in Figure 2c. Both SLs have the same ***a*** ^0^ ***a*** ^0^ ***c*** ^−^ octahedral rotation pattern,^[^ ^11^ ^]^ but the SL_5_ shows less tensile strain (***c*** = 4.013 Å) compared to SL_2_. The change of out‐of‐plane lattice constant suggest the occurrence of the strain relaxation, and the strain status of superlattice with different periods has been discussed in Figure S4, Supporting Information. The measured difference in crystal structure between SL_2_ and SL_5_, has an obvious effect on the materials properties.

**Figure 3:**
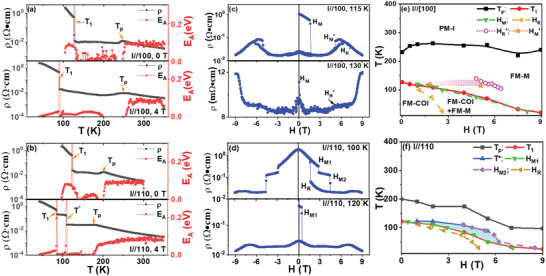
The resistivity‐temperature curve and calculated activation energy‐temperature curve (only the positive part) for SL_5_ on (001) LAO substrate, under different magnetic fields with a) I//[100] and b) I//[110]; the magnetoresistance curves at different temperatures with c) I//[100] and d) I//[110]; summarized magnetic field‐temperature phase diagram with e) I//[100] and f) I//[110]. The magnetic field is along out‐of‐plane directions.

With increasing temperature, there is one sharp down‐turn of resistivity (***T***_1_) in RT curve with ***I***//[100] for SL_5_ in **Figure** **3**a, one less than that of SL_2_; with ***H*** the melting of COI phase was boosted with decreasing ***T***_1_, which has similar trend as that of SL_2_. The MR curve was measured at different temperatures: At 115 K close to ***T***_1_, the ***H*** could melt the CO phase at ***H***_M1_ ≈1.6 T, and with increasing ***H*** it could back into high resistivity phase at $H_{R}^{\prime }$ ≈6.1 T, which was ascribed to the combing effect of variable range hopping (VRH) and the thermal fluctuation around the phase transition point.^[^ ^11^ ^]^ Then, with decreasing ***H***, the high resistivity state could be melted to low resistivity state at $H_{M}^{\prime }$ ≈5.1 T, however, it cannot return back to its initial high‐resistivity state with further decreasing ***H***, which suggests the coexistence of multiple phases.^[^ ^9^, ^26^ ^]^ At 130 K above ***T***_1,_ the film is at ferromagnetic metallic phase, and the resistivity gradually increase at high ***H*** (also labeled as $H_{R}^{\prime }$) due to the shrinkage of electronic orbital.^[^ ^27^ ^]^ One other factor inducing increasing resistivity with magnetic field is the Lorentz effect. In LSMO manganite,^[^ ^28^ ^]^ the estimated mean free path *l* ≈ 2 nm and the cyclotron orbit *r_c_* ≥ 6.8 µm, suggesting that the influence of the Lorentz effect in our case is negligible. Figure 3e summarizes the ***H‐T*** phase diagram: The dashed line is obtained from the MR curves, which reveals the coexistence of the FM‐M phase and FM‐COI phase. Above ***T***_1_, there is a tiny area (gradient pink color) between $H_{R}^{\prime }$ and $H_{M}^{\prime }$, which has different properties from FM‐M as discussed below.

With ***I***//[110] direction, the zero‐field *ρ*‐T curve of SL_5_ shows one down‐turn, and the magnetic field (***H*** > 0) could induce one additional down‐turn (***T*** ^*^) compared to that with ***I***//[100], which is similar to that in SL_2_ but with one fewer down‐turn. The change from SL_5_ to SL_2_ is studied by fabricating superlattice with three periods (SL_3_) on LAO substrate. The *ρ*‐T curves with ***I***//[110] of SL_3_ (Figure S5, Supporting Information) shows only one down‐turn at 0 T, and an additional down‐turn under high ***H***. These results indicate that the change process occurs abrupt between SL_2_ and SL_3_. The MR curves at different temperature also shows down‐turns of resistivity with increasing ***H***, which is summarized in the ***H‐T*** phase diagram of Figure 3f. With varying ***H***, there are some features with ***I***//[110]. First, compared to that with ***I***//[100], high ***H*** widens the temperature range of COI phase and increases the resistivity between ≈ ***T***_1_ (value for ***I***//[100] at 4 T) and ***T*** ^*^, which seems counterintuitive in manganite. The multiple melting of COI with different physical origins has been observed in other manganite work,^[^ ^29^ ^]^ and the anisotropic SOC is proposed to explain the anisotropic melting of COI phase.^[^ ^30^ ^]^ The metallic cluster in manganite tends to grow along the magnetic easy axis, and the anisotropic in‐plane strain could induce anisotropic transport properties via different percolation path. In current superlattices, the preferred occupancy of ***e***_g_ orbital 3*d*_r_^2^_‐z_^2^ has the main contribution to the conductivity. The *ρ*‐T curve indicates that the COI phase is easier (lower ***T***_1_) to melt into FM‐M phase along [110] direction without ***H***, suggesting that the SOC is stronger with enhanced coupling between neighboring Mn sites along this direction, although the lobes of the ***e***_g_ orbital (3*d*_r_^2^_‐z_^2^) are not directing at [110] direction. Such phenomenon has been reported in previous work.^[^ ^31^ ^]^ In the VRH process, the charge carrier transport is mediated by the spatial extension of the electronic orbitals, and orbital shrinkage would hinder the probability of charge hopping among different sites. The spatial extension of orbital is subject to magnetic field, which could cause the shrinkage of spatial distribution in the film plane for both 3*d*_r_^2^_‐z_^2^ and 3*d*_x_^2^_‐y_^2^ orbitals. Considering the relative higher occupancy of 3*d*_r_^2^_‐z_^2^ orbital and higher possibility of charge hopping along [110] direction, the orbital shrinkage induced by magnetic field would affect the charge hopping process along [110] direction obviously, which induces the widen temperature range of COI phase along [110] direction. The appearance of sharp down‐turn of resistivity is an overall effect of all conductive paths and a statistical outcome according to the percolation theory, with good stability as shown in Figure S6, Supporting Information. Besides the anisotropic SOC, the ordering of oxygen vacancy in the film plane might contribute to the anisotropic behavior as suggested by first‐principle simulation, but the measured polarization‐dependent XAS suggest that this effect is limited (Figure S7, Supporting Information). The second is that the relative change of resistivity in *ρ*‐T curve at each sharp down‐turn decreases compared to that without ***H***. Comparing the value of *E_A_*, ***H*** could lower the activation energy, 113 meV at 0 T and 82 meV at 4 T. According to VRH model, the activation energy is described^[^ ^32^ ^]^ as $E_{A}=\frac{3}{4{\pi d}^{3}N\left(E_{F}\right)}$, ***d*** is the distance between hopping sites, and N(E_*F*_) is the density of states at Fermi level (constant in Mott VRH). The decrease of activation energy under ***H*** suggests the increase of hopping distance, which may attenuate the effect of appearance of conducting bridge (appearance of down‐turn in resistivity) among clusters, and decrease the relative change of resistivity.

Careful comparison indicates that the ***T***_1_ for ***I***//[100] has the same trend as ***T***_1_ for ***I***//[110] with close value in SL_5_; What is interesting is that the $H_{R}^{\prime }$ curve in Figure 3e shows the same trend as ***T***_p_ from *ρ*‐T curves in Figure 3f with a close value, which suggests that the decrease of ***T***_p_ with ***H*** for ***I***//[110] is related to the ***H***‐dependent VRH process. The ***E***_A_‐***T*** curve under high ***H*** also shows temperature‐dependent above ***T***_p_, which could support the above argument that the transport mechanism above ***T***_p_ has more contributions, besides the nearest neighboring hopping. In Figure 3f, the ***T*** ^*^ exists with medium ***H***, either too high, and too low ***H*** cannot induce the appearance of ***T*** ^*^. The *H*_*M*2_ curve, which overlaps with ***T*** ^*^ partially, merges into ***T***_1_ curve with increasing ***H***, suggesting that ***H*** has a significant effect on the appearance of ***T*** ^*^. Between *H*_*M*1_ and *H*_*M*2_ in MR curve, the resistivity decreases linearly with magnetic field, signaling the quantum interference (QI) effect during VRH process in already‐formed COI phase. According to the above discussion, in the area enclosed by ***T***_1_ (or *H*_*M*1_), ***T*** ^*^ and *H*_*M*2_ curves of Figure 3f (blue color), corresponding to the widened temperature range of COI phase. In the VRH process, the transport of the charge carrier is mediated by the spatial extension of the electronic orbitals, and the ***H***‐induced orbital shrinkage would hinder the probability of charge hopping among different sites and increase the resistivity. For ***I***//[110] with stronger SOC, the probability of charge hopping is sensitive to the magnetic field‐induce orbital shrinkage, resulting in widen temperature range of COI phase (blue area in Figure 3f) and the observed T*. While for ***I***//[100], no such T* was observed, suggesting the effect of magnetic field‐induce orbital shrinkage is limited.

To sum up, the condition of ***I***//[100] and strong ***H*** could induce a high resistivity phase (gradient pink color in Figure 3e) close to the zero‐field phase transition point (***T***_1_), around which the effect of thermal fluctuation would be important and might induce the recovery of high resistivity phase from low resistivity phase in the VRH process. With ***I***//[110] strong ***H*** could widen the temperature range of high resistivity area (blue color in Figure 3f) below zero‐field phase transition point (***T***_1_). The anisotropic behavior should come from the sensitivity of charge hopping to thermal fluctuation, and the orbital spatial distribution along different directions.

For SL_2_, ***T***_1_ and ***T***_2_ for ***I***//[100] also has the same trend as ***T***_1_ and ***T***_2_ for ***I***//[110] in Figure 1e,f, respectively. Considering the similarity between SL_2_ and SL_5_, the same mechanism has been proposed for ***T*** ^*^ in SL_2_. The persistent transition ***T***_1_ and ***T***_2_ exist along both in‐plane [100] and [110] directions, and the field‐induced emergent transition ***T*** ^*^ only appears along [110]. The sharp down‐turn at ***T***_1_ and ***T***_2_ comes from the collapse of the COI phase when the kinetic energy of the charge carriers through double exchange interaction dominates over the COI stability, and the emergent conducting path forms along [110] direction, which could be induced by magnetic and then ***T*** ^*^ appears. Considering that the activation energy *E_A_* of ***T*** *_1_*, ***T*** *_2,_* and ***T*** * are similar and ***T*** * only appears for ***I***//[110], the appearance of ***T*** *_1_* and ***T*** *_2_* without magnetic field is due to the spatial inhomogeneity in as‐grown film, and appearance of additional ***T*** * with the magnetic field is ascribed to the additional spatial inhomogeneity induced by application of strong magnetic field, arising from the competing between charge hopping in double‐exchange process and the variable‐range hopping. With larger tensile strain along the out‐of‐plane direction for SL_2_ (***c*** = 4.036 Å) compared to SL_5_ (***c*** = 4.013 Å), the COI phase exists in a wider temperature range in SL_2_, suggesting that the out‐of‐plane tensile strain could stabilize the COI phase.^[^ ^33^, ^34^ ^]^ In order to verify the strain effect on the COI phase, superlattice with 10 periods (SL_10_) on LAO substrate was fabricated, and strain relaxation is observed. The *ρ*‐T curve also shows insulator behavior at low temperatures, but no abrupt jump occurs with relatively low resistivity compared to that of SL_2_ and SL_5_. The measured resistivity is the averaged results of the whole superlattice, and the gradual increase of resistivity with decreasing temperature (Figure S4, Supporting Information) suggests that the relaxed part in SL_10_ has less contribution to the feature of COI phase. In addition, superlattice structures same as SL_2_ and SL_5_ have been fabricated on STO substrate. These superlattices experience in‐plane tensile strain and out‐of‐plane compressive strain, and their *ρ*‐T curves do not show any COI phase (Figure S8, Supporting Information). These results indicate that the out‐of‐plane tensile strain is crucial for the formation of the insulating COI phase. The effect of LSMO thickness in one period on the superlattices’ properties have been discussed in Figure S9, Supporting Information.

**Figure 4:**
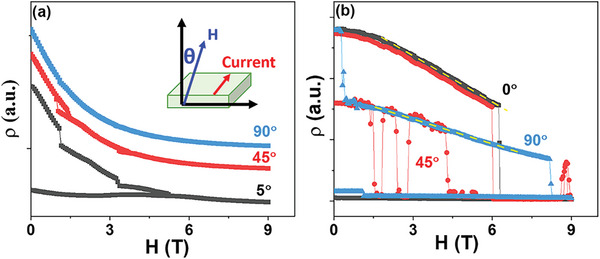
The magnetoresistance curve with magnetic field rotating from out‐of‐plane to in‐plane directions for a) SL_5_ at 115 K and b) SL_2_ at 170 K. The inset is the illustration of measurement configuration. For SL_5_, the magnetoresistance curve with ***θ*** = 0˚is shown in the upper part of Figure 3c.

With a large anisotropy of crystal structure along the in‐plane and out‐of‐plane directions, the angular magnetoresistance (AMR) was also obtained with ***H*** rotation from [001] to [010] directions, and the current was kept along the [100] direction, as shown in **Figure** **4**a,b. For SL_5_ with ***H*** up to 9 T, the resistivity ***ρ*** (9T) is the same for ***H*** along different directions, but the process is quite different. For ***H*** close to [001] direction (*θ* = 5˚), there are several jumps with increasing ***H***, and the resistivity cannot back to its initial value with decreasing ***H***, while there is no jumps for ***H***//[010] (*θ* = 90˚) and the resistivity could back to its initial value. The continuous change of resistivity without sharp jumps means a small relative change of resistivity with each step length of magnetic field. The measurements of crystal structure indicates a larger out‐of‐plane lattice constant ***c*** than in‐plane ***a***, and the electrons tend to occupy $3d_{z^{2}-r^{2}}$ orbital.^[^ ^35^, ^36^ ^]^ The anisotropic orbital ordering might cause anisotropic melting of the insulator phase due to the intimate coupling between spin and orbital degree of freedom.^[^ ^9^ ^]^ In the current work, the orbital ordering would induce anisotropic response of the SOC and double‐exchange interaction to the magnetic field. Along the direction with a high Mn‐O orbital integral, the magnetic field would increase the hopping probability of electron largely that could enhance the SOC and double‐exchange interaction along out‐of‐plane directions. Along other directions, the effect of magnetic field on hopping probability of electron would be weakened by a limited orbital integral. With ***H*** rotating from the [001] to [010] direction, the effect of ***H*** on enhancing magnetic interactions decrease, which would weaken the tendency of sharp melting of COI phase.^[^ ^9^ ^]^ The melting of CO phase would therefore take place gradually without a sharp down‐turn for ***H***//[010] as observed. This also could explain the difference between *θ* = 5˚ and *θ* = 0˚ (upper of Figure 3c): the strong SOC along *θ* = 0˚ direction tends to cause the collapse of COI phase in lower magnetic field with large jump of resistivity.

For SL_2_, the AMR shows a sharp change of resistivity with ***H*** along different directions as shown in Figure 4b. The MR experiences one big jump for ***H***//[001], and it experiences more than one jump for ***H***//[010]. A strong magnetic field is required to melt the COI phase in SL_2_ due to its high stability, and the resistivity under ***H*** is dominated by the QI effect in the measured range of magnetic field, revealed as the linear dependence of resistivity on ***H*** and labeled as yellow dashed line in Figure 4b. However, the slope, which is proportional^[^ ^13^ ^]^ to the Mott's characteristic temperature ***T***_0_, changes with direction of ***H***. Comparing the MR at 90° and 0°, the lower slope indicates a smaller ***T***_0_ and activation energy^[^ ^37^ ^]^ *E_A_* with ***H***//[010]. Similar to the *ρ*‐T curves under high ***H***, the decrease of ***E***_***A***_ would increase the hopping distance and attenuate the effect of appearance of conducting bridge between clusters, inducing a gentler change of resistivity at each jump as observed. However, with ***H*** 45° to the normal direction, the MR shows quite a different behavior: multiple down‐turn appears, and fluctuation between different states appears with decreasing ***H***, which should correspond to the transition between different conditions. The AMR of both SL_2_ and SL_5_ with similar trend indicates that by changing the strength of different interactions, the melting path of COI phase could be largely modulated.

## 4 Conclusion

The La_0.7_Sr_0.3_MnO_3_/SrTiO_3_ superlattice with different periods were fabricated on (001) LaAlO_3_ (LAO) substrate, and the melting process was investigated. For both SL_2_ and SL_5_, the magnetic field‐temperature phase diagram shows anisotropy for ***I***//[100] and ***I***//[110] with the magnetic field normal to the film plane. The response of the VRH process to magnetic field is proposed to cause the additional step for ***I***//[110], and the anisotropy comes from the in‐plane anisotropic hopping probability of charge carrier. With magnetic field rotating from the out‐of‐plane to the in‐plane directions, the preferred occupancy of $3d_{z^{2}-r^{2}}$ causes decreasing spin‐orbital coupling and weakens the tendency of the COI collapse, which causes gentler melting of the COI phase in magnetoresistance curve. This work indicates that the melting path of COI phase could be modulated by changing the strength of different couplings.

## Conflict of Interest

The authors declare no conflict of interest.

## Supporting information

Supporting Information

## Data Availability

The data that support the findings of this study are available from the corresponding author upon reasonable request.
