# Supplementary material for: Anisotropic Melting Path of Charge‐Ordering Insulator in LSMO/STO Superlattice
Source: Adv Sci (Weinh). 2022 Dec 3;10(4):2203933. doi: 10.1002/advs.202203933 (PMC9896059; doi:10.1002/advs.202203933)
Supplement: Supplementary file 1 — Supporting Information [file ADVS-10-2203933-s001.pdf]

ADVANCED SCIENCE
Open Access

Supporting Information

for *Adv. Sci.*, DOI 10.1002/advs.202203933

Anisotropic Melting Path of Charge-Ordering Insulator in LSMO/STO Superlattice

Bangmin Zhang, Ping Yang, Jun Ding, Jingsheng Chen* and Gan Moog Chow**

Supplementary Materials

for

Anisotropic melting path of charge-ordering insulator in LSMO/STO superlattice

Bangmin Zhang,[1,*] *Ping Yang,*[2] *Jun Ding,*[3] *Jingsheng Chen,*[3]

Gan Moog Chow,[3,*]

[1]Guangdong Provincial Key Laboratory of Magnetoelectric Physics and Devices, Centre for Physical Mechanics and Biophysics, School of Physics, Sun Yat-sen University, Guangzhou 510275, China.

[2]Singapore Synchrotron Light Source (SSLS), National University of Singapore, 5 Research Link, 117603, Singapore.

[3]Department of Materials Science & Engineering, National University of Singapore, 9 Engineering Drive 1, 117576, Singapore.

*Corresponding authors: zhangbm5@mail.sysu.edu.cn, msecgm@nus.edu.sg

Outline

S1: Transport properties of LSMO single layer on LAO substrate

S2: Magnetic properties

S3: Transport model

S4: Properties of SL_{10} on LAO substrate

S5: Transport properties of SL_3 on LAO substrate

S6: Stability of transport properties

S7: In-plane ordering of oxygen vacancy

S8: Transport properties of superlattice on (001) STO substrate

S9: Effect of LSMO thickness

S1: Transport properties of LSMO single layer on LAO substrate

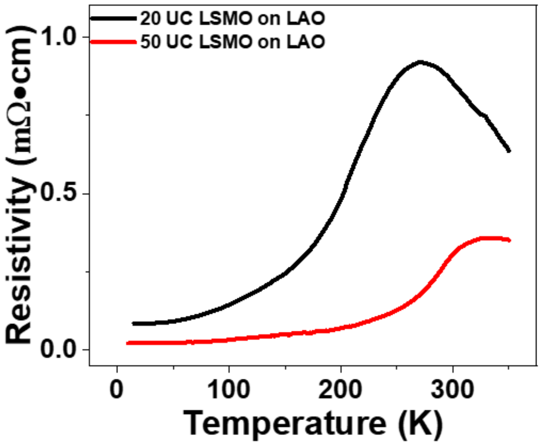

Figure S1: Resistivity-temperature curves for 20 UC and 50 UC LSMO films on LAO substrate.

S2: Magnetic properties

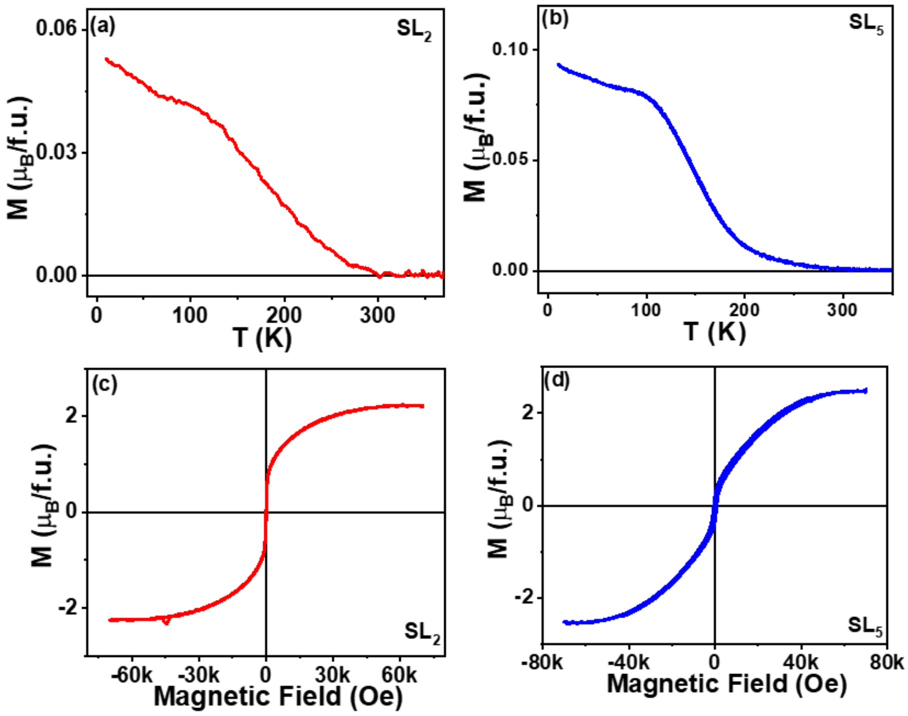

Figure S2: The MT curves at in-plane 50 Oe field for (a) SL_2 and (b) SL_5; the magnetic hysteresis loop at 10 K for (c) SL_2 and (d) SL_5; these results indicate that the superlattices are ferromagnetic at low temperatures.

S3: Transport model

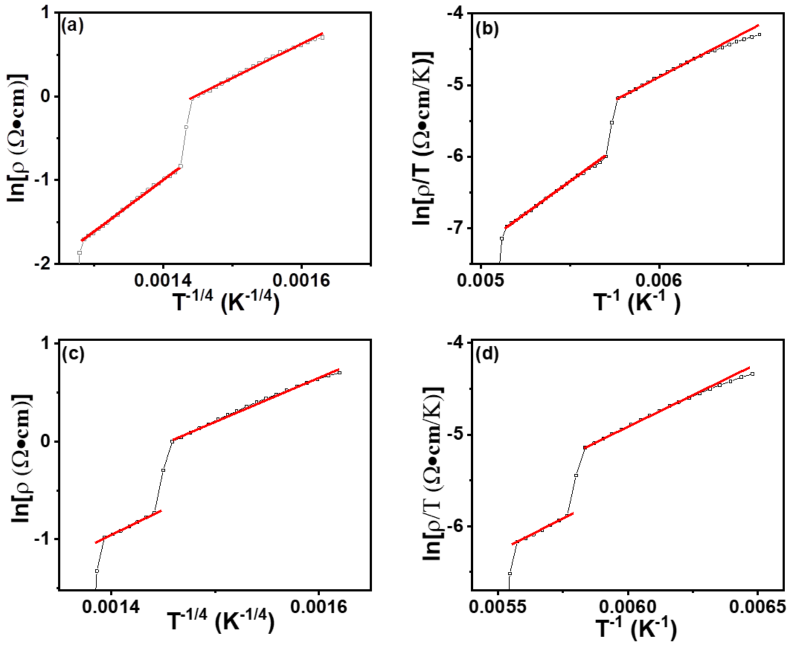

Fig. S3-1: The resistivity-temperature curve in ln(ρ) vs $T^{-1/4}$ and ln(ρ/T) vs T^{-1} plots, for SL_2 along the [100] (a-b) and [110] (c-d) directions.

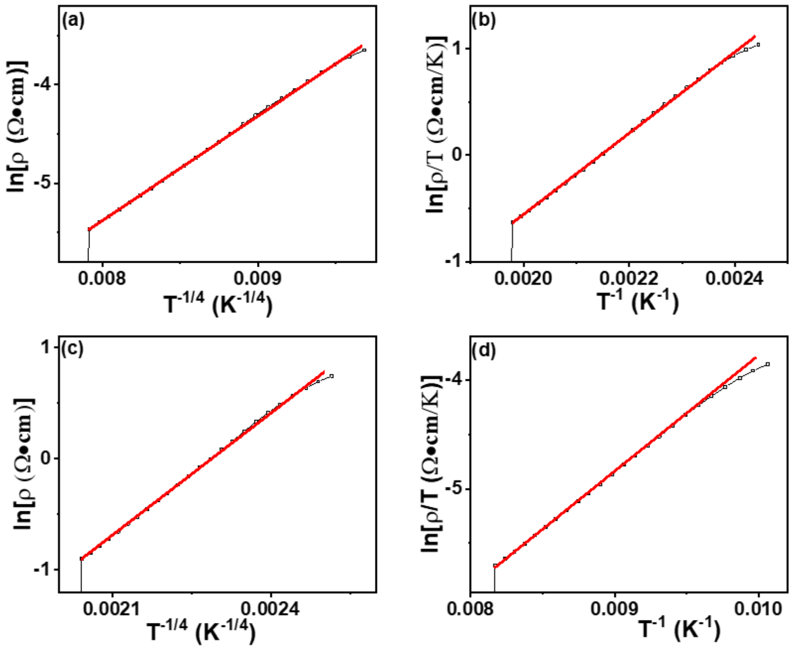

Fig. S3-2: The resistivity-temperature curve plots in ln(ρ) vs $T^{-1/4}$ and ln(ρ/T) vs T^{-1} plots, for SL_5 along [100] (a-b) and [110] (c-d) directions.

For VRH model $\rho = \rho_0 \exp\left[(T_0/T)^{1/4}\right]$, and for small polaron hopping model $\rho = \rho_\alpha T exp(E_p/k_B T)$. Fig.S3-1 shows the ln(ρ) vs $T^{-1/4}$ and ln(ρ/T) vs T^{-1} plots for SL_2. Comparing the Fig.S3-1(a) and Fig.S3-1(b), the ln(ρ) vs $T^{-1/4}$ plot shows a better linearity,

which suggests that the VRH model could better describe the transport process along the [100] direction. Simialr result applies for the resistivity-temperature curve along the [110] direction. Fig. S3-2 shows the resistivity-temperature curve plots along the [100] and [110] directions for SL_5, which also could be described more properly by VRH model.

S4: Properties of SL_{10} on LAO substrate

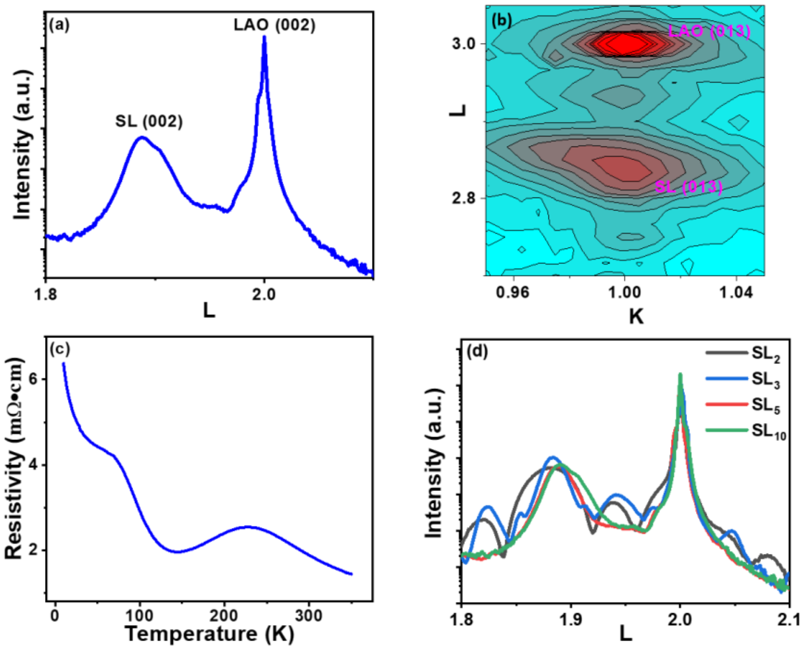

Fig. S4: (a) The ***L*** scan, (b) (013) reciprocal space mapping and (c) transport property for SL_{10} on LAO substrate. Strain relaxation occurs and no sharp jump in resistivity is measured. (d) is the comparison of ***L*** scan for superlattice with different periods.

The ***L*** scan of SL_2, SL_3, SL_5 and SL_{10} superlattices was compared. The corresponding peak position (out-of-plane lattice constant) is $\boldsymbol{L}$ = 1.882 ($\boldsymbol{c}$ = 4.029 Å) for SL_2, $\boldsymbol{L}$ = 1.884 ($\boldsymbol{c}$ = 4.023 Å) for SL_3 and $\boldsymbol{L}$ = 1.889 ($\boldsymbol{c}$ = 4.013 Å) for SL_5; while for SL_{10} two peaks appears with larger (smaller) ***L*** (***c***) compared to all other superlattices, indicating that the strain relaxation dominates the structure. LSMO/STO superlattices fabricated on LAO substrate is under in-plane compressive strain, and out-of-plane tensile strain conditions. With occurrence of strain relaxation, the out-of-plane strain should gradually shift back to its bulk value. With the increase of superlattice periods, the out-of-plane decreases, which follows the trend with the existence of strain relaxation. With the increase of periodic number (total thickness) of superlattice, strain relaxation occurs and the features of COI phase weaken, which is consistent with the that the out-of-plane tensile strain (in-plane compressive strain) is important to the formation of the COI phase.

S5: Transport properties of SL_3 on LAO substrate

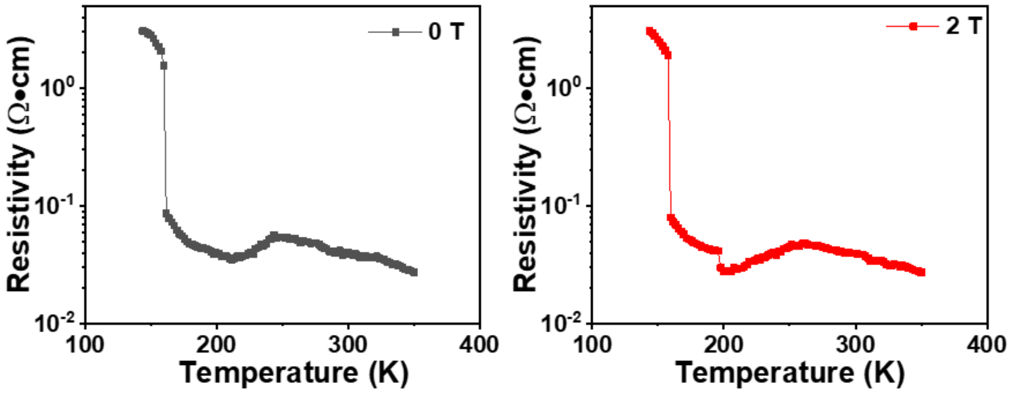

Figure S5: Resistivity-temperature curves along the [110] direction for SL_3 on LAO substrate at (a) 0 T and (b) 2 T.

S6: Stability of transport properties

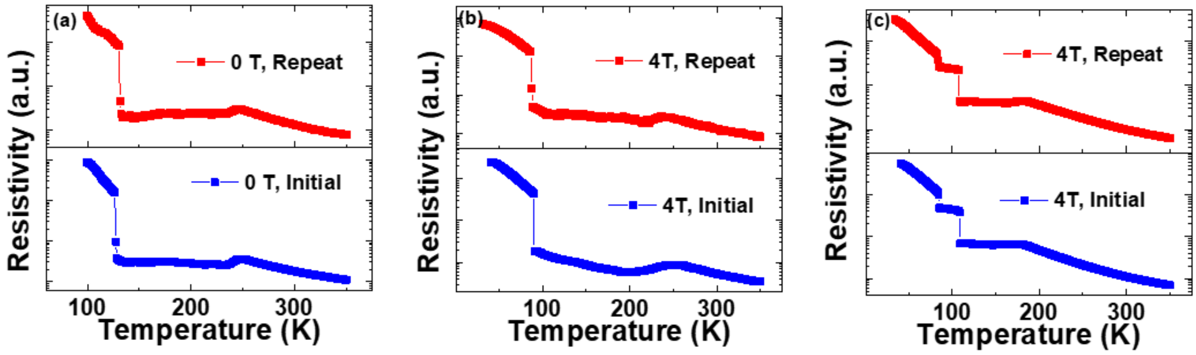

Figure S6: Remeasured Resistivity-temperature curves with (a-b) I//[100] and (c)) I//[110]. The transport properties show good stability.

S7: In-plane ordering of oxygen vacancy

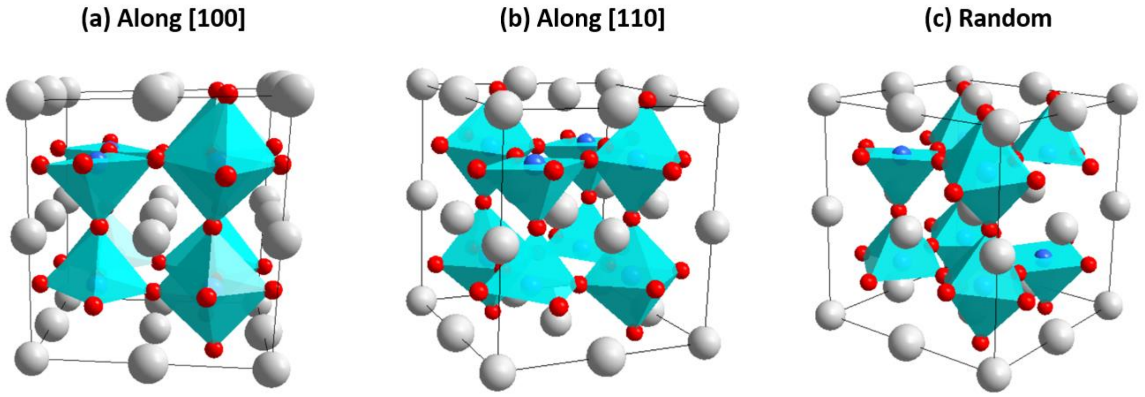

Figure S7: Simulation configuration with different ordering of oxygen vacancy. The white ball is the La/Sr atoms, the red is the O atoms and the blue is the Mn atoms.

The first principle simulation has been conducted to study the selective location of oxygen vacancies on (001) plane. Three cases, with two oxygen vacancy along [100] and [110] direction on the same (001) plane, respectively, and random distribution at two (001) planes, have been investigated by VASP simulation. Comparing the total energy, the oxygen vacancy along [110] direction has the highest energy (-295.29 eV) and that along [100] direction has the lowest energy (-295.37 eV), and the energy of random distribution (-295.35 eV) is between them but close to that with oxygen vacancy along [100] direction. With the oxygen vacancy located along [100] direction, the possibility of charge hoppling along this direction would be hindered considering that the oxygen generally severs as the bridge of the charge hopping. Hence, the selective location of oxygen vacancies on (001) plane might contribute to the observed in-plane anisotropic properties by affect the probability of charge hopping. However, the polarization-dependent XLD measurement shows on obvious difference with electric field of X-ray along in-plane [100] and [110] directions, which suggest that the effect of selective location of oxygen vacancies on (001) plane in current work should be limited.

S8: Transport properties of superlattice on (001) STO substrate

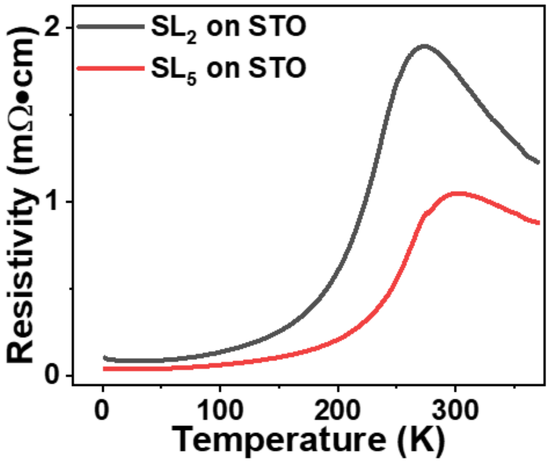

Figure S8: Resistivity-temperature curve of SL_2 and SL_5 on LAO substrate.

S9: Effect of LSMO thickness

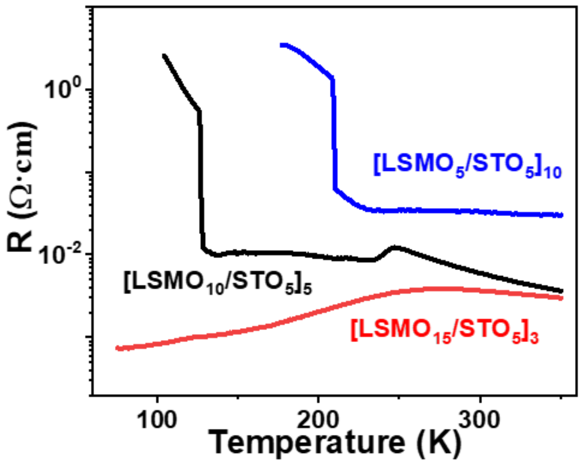

Figure S9: ρ-T curves of superlattice with different LSMO thickness in one period (Some datum retrieved from *Journal of Materials Chemistry A, 9 (2021) 26717-26726*).

The ρ-T measurement indicates that [LSMO$_{15}$/STO$_5$]$_3$ superlattice do not show any COI feature, but COI appears in [LSMO$_5$/STO$_5$]$_{10}$ superlattice and could sustain up to ~ 210 K, higher than that in [LSMO$_{10}$/STO$_5$]$_5$ superlattice. Compared to [LSMO$_{10}$/STO$_5$]$_5$, more (less) LSMO/STO interfaces exit in [LSMO$_5$/STO$_5$]$_{10}$ superlattice ([LSMO$_{15}$/STO$_5$]$_3$) with enhanced (weakened) COI feature. Larger ratio of LSMO around the LSMO/STO interface in [LSMO$_5$/STO$_5$]$_{10}$ superlattice was affected by the STO/LSMO interfacial coupling, resulting in enhanced feature of COI phase.

With the decrease of period number (total thickness) of [LSMO$_{10}$/STO$_5$]$_n$ superlattice, the effect of the lattice-mismatch strain by substrate on its structure increases, which enhances the feature of COI phase. While with changing the thickness of LSMO in the period, the trend is different. The total thickness of ([LSMO$_{15}$/STO$_5$]$_3$ superlattice is 60 UC, which is smaller than that of [LSMO$_5$/STO$_5$]$_{10}$ superlattice (100 UC), and the effect of lattice-mismatch strain by substrate on its structure should be more pronounced. However, the [LSMO$_{15}$/STO$_5$]$_3$ superlattice has no feature of COI phase, while [LSMO$_5$/STO$_5$]$_{10}$ superlattice has obvious feature of COI phase. These phenomena could be understood as below: The coupling STO/LSMO interface induces the formation of COI phase, more ratio of LSMO affected by this coupling, the stronger the feature of COI phase. In the case with

changing LSMO thickness in one period of LSMO/STO superlattice, the dominating factor is the LSMO/STO interfacial coupling (stronger interfacial coupling in $[LSMO_5/STO_5]_{10}$). With the fixed ratio of LSMO affected by interfacial coupling, corresponding to changing the periodic number of superlattice, the in-plane compressive strain tends to stabilize the COI phase, and with strain relaxation in superlattice of high number of periods, the feature of COI phase weakens.
